# Expression of HIF-1α and VEGF in feline mammary gland carcinomas: association with pathological characteristics and clinical outcomes

**DOI:** 10.1186/s12917-020-02338-y

**Published:** 2020-05-06

**Authors:** Bo Chen, Susanne Je-Han Lin, Wen-Ta Li, Hui-Wen Chang, Victor Fei Pang, Pei-Yi Chu, Chin-Cheng Lee, Hiroyuki Nakayama, Ching-Ho Wu, Chian-Ren Jeng

**Affiliations:** 1 Graduate Institute of Molecular and Comparative Pathobiology, National Taiwan University, Taipei, Taiwan; 2 Institute of Veterinary Clinical Science, School of Veterinary Medicine, National Taiwan University, Taipei, Taiwan; 3 Department of Pathology, Show Chwan Memorial Hospital, Changhua, Taiwan; 4 Department of Pathology, Shin Kong Wu Ho-Su Memorial Hospital, Taipei, Taiwan; 5 Department of Pathology, The University of Tokyo, Tokyo, Japan

**Keywords:** Feline, Mammary gland carcinoma, Hypoxia-inducible factor 1 alpha (HIF-1α), Vascular endothelial growth factor (VEGF)

## Abstract

**Background:**

The microenvironment within solid malignant tumors, including feline mammary gland carcinomas (FMGCs), is commonly hypoxic, possibly due to the lack of functional blood vessels in rapidly proliferating neoplastic tissue. Malignant cells can undergo genetic and adaptive changes that prevent them from dying due to oxygen deprivation through expressions of hypoxia-inducible factor 1 alpha (HIF-1α) and vascular endothelial growth factor (VEGF). Therefore, HIF-1α and VEGF are ideal biomarkers for cancer therapy and prognostic evaluation. The aims of this study were to evaluate the expression of HIF-1α and VEGF in feline mammary carcinomas and analyze their correlations with clinical and pathological factors, such as clinical stage, histologic grading, regional metastasis, and overall survival rate.

**Results:**

Paraffin-embedded tissue samples collected from 72 cats with FMGCs were retrospectively studied. Histologic pattern and histologic grading (Elston and Ellis grading system) of these FMGCs were determined. Our data indicated that grade II tubulopapillary carcinomas (43/72, 59.7%) prevailed in this study, and most FMCGs showed apparent necrosis, squamous metaplasia, and intratumoral stromal response. According to the results of immunohistochemical (IHC) stainings performed in tissue microarrays (TMAs), HIF-1α and VEGF overexpressions were respectively noted in 69.4% (50/72) and 77.8% (56/72) of FMGC cases. Chi-square test showed no correlation of HIF-1α overexpression with clinical and pathological factors. VEGF overexpression was significantly correlated with histologic pattern (*p* = 0.021), stromal response (*p* = 0.048), squamous metaplasia (*p* = 0.001), and lymphovascular invasion (*p* = 0.007). However, neither HIF-1α nor VEGF overexpression was correlated with histologic grading and metastasis. Of 38 cats with 1-year follow-up, IHC stainings of HIF-1α and VEGF were performed on whole tissue sections. The results showed that overexpression of HIF-1α was significantly correlated with the overall survival rate (*p* < 0.05) (log-rank test), whereas there was no significant correlation between VEGF overexpression and overall survival rate.

**Conclusions:**

This study suggests that the overexpression of HIF-1α may indicate poor prognosis/overall survival rate in cats with FMGCs. Developing compounds that inhibit HIF-1α may be a potential approach to FMGC treatment.

## Background

Mammary neoplasia is the third most common neoplasia affecting female cats [1]. Feline mammary gland carcinomas are frequently reported with local recurrence and distant metastasis, resulting a high mortality rate [2]. Based on clinical and pathological similarities between FMGCs and human breast cancers, FMGCs have been considered an excellent animal model for the human counterpart [3, 4]. Several clinical and pathological parameters are prognostic for both human breast cancers and FMGCs [5]. Immunohistochemical (IHC) staining is emerging as a method for understanding the molecular pathogenesis of FMGCs, and such understanding may further contribute to FMGC therapies and comparable research on human breast cancers.

A hypoxic microenvironment is frequently present in many solid malignant tumors, including FMGCs. It may be due to the lack of functional blood vessels in rapidly proliferating neoplastic tissue [6]. A prolonged and severely hypoxic condition is usually fatal for neoplastic cells, and thus they will undergo genetic and adaptive changes to accommodate the hypoxic condition [7, 8]. Furthermore, several previous studies have demonstrated that an insufficient level of cellular oxygen is significantly correlated with carcinogenesis, cancerous invasion/metastasis and cell death [8–10].

Hypoxia-inducible factor 1-alpha (HIF-1α) plays a crucial role in neoplastic cells responding to low-oxygen tension in a variety of physiologic processes, including angiogenesis, tumorigenesis and metastasis [11, 12]. After neoplastic cells are exposed to hypoxia, the amount of HIF-1α heterodimerization with the HIF-1β protein (ARNT) rapidly increases, leading to increases in the HIF-1 protein in the nucleus/cytoplasm [13, 14]. The amount of HIF-1α protein in the nucleus indicates the functional activity of the HIF-1 complex, which may alter the genetic transcription involving angiogenesis, including vascular endothelial growth factor (VEGF) [14]. VEGF overexpression is correlated with the degree of tumor vascularization, tumor progression and diagnosis in many human breast cancers [15]. Furthermore, HIF-1α and VEGF are novel biomarkers for cancer therapy and have led to a great shift in drug development.

In veterinary pathology, although VEGF has been documented as a good prognostic marker for FMGCs, some previous studies have demonstrated inconsistent findings [16–19]. Furthermore, no studies on the overexpression of HIF-1α in FMGCs have been reported. Therefore, the aims of the present study were (1) to construct FMGC tissue microarrays (TMAs) from the archives for the period of January 2015 to December 2015 at the Graduate Institute of Molecular and Comparative Pathobiology, National Taiwan University, (2) to immunohistochemically investigate the overexpression of HIF-1α and VEGF in FMGCs, and (3) to evaluate whether the overexpression of VEGF and/or HIF-1α is significantly correlated with clinical and pathological factors, such as clinical stage, histologic grading, regional metastasis, and overall survival rate.

## Results

### Clinical and pathological information on the cases of FMGCs

Of the 72 feline mammary carcinoma tissues obtained, 45 cases were from spayed females and 3 were from males (1 intact and 2 castrated). The median age was 11.6 ± 3.04 (4–19). No patients had history of chemotherapy or radiotherapy during the survey. On the basis of histologic pattern, tubulopapillary carcinoma was the most common pattern of FMGCs (52/72, 72.2%), followed by solid carcinoma (10/72, 13.9%), cribriform (6/72, 8.3%), and micropapillary invasive carcinoma (4/72, 5.6%). Lymphovascular invasion was found in 48.6% (35/72) of the cases. The results of histologic grading demonstrated that grade II FMGCs (59.7%, 43/72) prevailed in this study. Sixteen of the 26 submitted lymph nodes had metastasis. In addition, most cases had obvious necrosis, squamous differentiation, and intratumoral stromal response.

### Overexpression and pattern of HIF-1α and VEGF in FMGCs

**Fig. 1:**
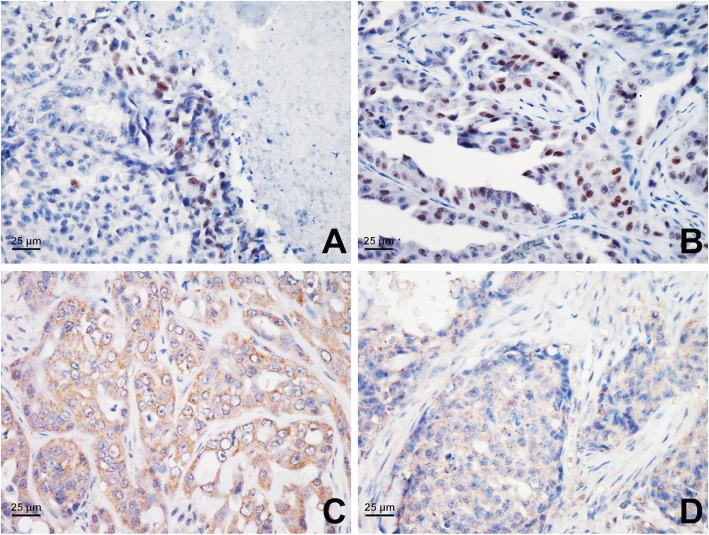
HIF-1 α and VEGF immunohistochemical expression in feline mammary gland carcinomas (FMGCs). **a.** Perinecrotic pattern of HIF-1 α expression, **b.** Diffuse pattern of HIF-1α expression, **c.** Cytoplasmic VEGF expression, **d.** Cytoplasmic VEGF expression in neoplastic epithelial cells and mesenchymal stromal cells of FMGCs. Magnification, × 40 (panels **a**-**d**)

HIF-1α overexpression was detected in 69.4% (50/72) of the FMGC cases by using constructed TMAs. The positive signals of HIF-1α in neoplastic cells were mainly nuclear, but mixed nuclear/cytoplasmic positivity was occasionally noted. Neoplastic cells with HIF-1α overexpression were scattered throughout the neoplasm and frequently observed nearby the necrosis (Fig. 1a, b). VEGF overexpression was observed in 77.8% (56/72) of the FMGC cases. Cytoplasmic VEGF positive signals were noted in neoplastic cells and occasionally observed in stromal cells (Fig. 1c, d). IHC stainings of HIF-1α and VEGF from 38 FMGC cases with 1-year follow-up were performed on whole tissue sections, and overexpressions of HIF-1α and VEGF were noted respectively in 78.9% (30/38) and 89.5% (34/38) of the FMGC cases.

### Correlations between HIF-1α/VEGF overexpression and clinical/pathological factors

The correlations between HIF-1α/VEGF overexpression and clinical/pathologic characteristics are summarized in Table 1. There were no significant correlations between HIF-1α expression and clinical/pathological factors. VEGF overexpression was significantly correlated with histologic pattern (*p* = 0.021, Chi-square), stromal response (*p* = 0.048, Chi-square), squamous differentiation (*p* = 0.001, Chi-square), and lymphovascular invasion (*p* = 0.007, Chi-square). VEGF overexpression was observed in all solid carcinomas (*n* = 10). VEGF overexpression was not correlated with other clinical and pathological parameters, including age, reproductive status, clinical stage, tubule formation, mitotic count, necrosis, lymph node metastasis, and histologic grading. Spearman analysis indicated that the HIF-1α overexpression was not statistically correlated with VEGF overexpression (*p* = 0.057, r = 0.226) in FMGCs.

**Table 1:** Association between HIF-1α or VEGF and clinical/pathological factors by using TMAs

Factors	No. of patients	HIF-1α		VEGF	
		Overexpression	*p* value	Overexpression	*p* value
Age					
< 12 y	32	19	ns	24	ns
≥ 12 y	40	31		32	
Reproductive status					
Intact female	24	13	ns	18	ns
Spayed female	45	34		36	
Intact male	1	1		1	
Castrated male	2	2		1	
Clinical stage					
I + II	34	20	ns	20	ns
III + IV	35	28		34	
Pattern					
Tubulopapillary	52	37	ns	41	0.021*
Solid	10	7		10	
Cribriform	6	4		2	
Invasive micropapillary	4	2		3	
Tubule formation					
>75%	39	27	ns	28	ns
10–75%	22	17		19	
<10%	11	6		9	
Mitotic count					
≤ 20	36	25	ns	29	ns
> 20	36	25		27	
Necrosis					
≤ 25%	32	21	ns	26	ns
> 25%	40	29		30	
Stromal response					
Mild	11	6	ns	6	0.048*
Peritumoral	12	10		8	
Intratumoral	49	34		42	
Squamous differentiation					
≤ 5%	27	17	ns	15	0.001*
> 5%	44	34		41	
Lymphovascular invasion					
Absent	37	17	ns	24	0.007*
Present	35	31		32	
Lymph node metastases					
Absent	10	6	ns	6	ns
Present	16	10		14	
EE grading system					
Grade 1	18	13	ns	14	ns
Grade 2	43	31		31	
Grade 3	11	6		11	
REE grading system					
Grade 1	40	29	ns	27	ns
Grade 2	28	19		25	
Grade 3	4	2		4	
Novel grading system					
Grade 1	5	4	ns	3	ns
Grade 2	30	23		20	
Grade 3	37	23		33	

*, *p* < 0.05

*ns* Not significant

### Correlation between pathological factors and overall survival rate

**Fig. 2:**
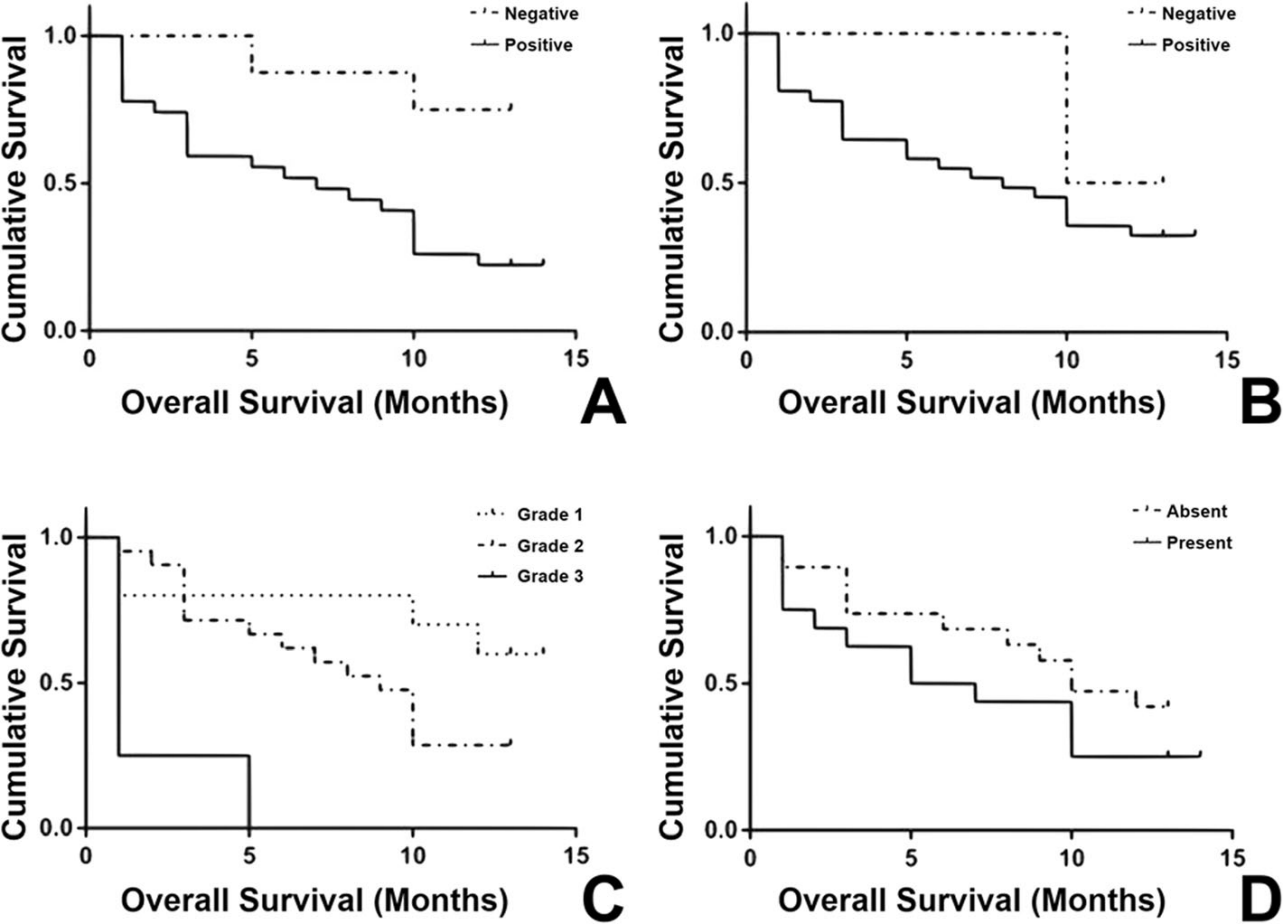
Kaplan-Meier curves of overall survival rate for 38 cats with mammary gland carcinoma using whole tissue sections. **a.** Cats with HIF-1 α overexpression had a significantly lower overall survival rate compared with those with HIF-1 α without overexpression. **b.** VEGF overexpression was not correlated with the overall survival rate of cats with FMGCs. **c.** Elston and Ellis grading system was significantly correlated with overall survival rate. **d.** No significant correlation between lymphovascular invasion and lower overall survival rate

Thirty-eight of the 72 cases had 1-year follow-up. Twelve patients were still alive and 26 were dead. Therefore, the 1-year overall survival rate for all patients was 31.6% (12/38). In this study, the Elston and Ellis (EE) and Revised Elston and Ellis (REE) grading systems had prognostic significance for FMGCs (*p* = 0.001, log-rank test, Fig. 2c). Necrosis (*p* = 0.035, log-rank test), stromal response (*p* = 0.030, log-rank test) and squamous metaplasia (*p* = 0.015, log-rank test) were significantly associated with prognosis. However, there was no significant correlation between lymphovascular invasion and overall survival rate (*p* > 0.05, log-rank test, Fig. 2d).

### Correlation between HIF-1α/VEGF overexpressions and overall survival rate

HIF-1α and VEGF overexpressions were demonstrated in 65.8% (25/38) and 76.3% (29/38) of the FMGCs by using TMAs. Furthermore, the IHC stainings of HIF-1α and VEGF on whole tissue sections indicated HIF-1α and VEGF overexpressions in 78.9% (30/38) and 89.5% (34/38) of the FMGCs, respectively.

There was a significant correlation between HIF-1α overexpression and overall survival rate. When whole tissue sections were used, the 1-year overall survival rates for FMGC cases with and without HIF-1α overexpression were respectively 80% (24/30) and 25% (2/8) (*p* = 0.033, log-rank test, Fig. 2c), but no significant correlation between HIF-1α overexpression and OS rate was found with TMAs (*p* = 0.086, log-rank test). The 1-year overall survival rates for FMGC cases with and without VEGF overexpression were 70.0% (24/34) and 40.0% (2/4) in the whole sections. No significant correlation between VEGF overexpression and overall survival rate was found either by TMAs (*p* = 0.617, log-rank test) or whole tissue sections (*p* = 0.285, log-rank test, Fig. 2d).

## Discussion

Several studies have demonstrated that tumor hypoxia through activation of angiogenesis assists in tumor progression and metastasis, which also makes solid tumors resistant to radiation and chemotherapy [20]. HIF-1α is a marker of hypoxia, which activates gene encoding glucose transporters, glycolytic enzymes, and VEGF [14]. It is widely accepted and confirmed in humans that HIF-1α causes the angiogenic switch via VEGF [14]. This retrospective study evaluated the overexpressions of HIF-1α and VEGF in FMGCs and investigated the correlations among the overexpressions of HIF-1α/VEGF, clinical/pathological factors, and clinical outcomes.

HIF-1α has been found to be overexpressed in a variety of human cancers, such as colon, breast, gastric, lung, skin, ovarian, pancreatic, prostate, and renal carcinomas [21–23]. In humans, the amount of HIF-1α is higher in primary and metastatic neoplasms than in the respective non-neoplastic tissues. To the best of our knowledge, this is the first IHC study of HIF-1α in FMGCs. Our study demonstrated that 69.4% (50/72) of the FMGCs had HIF-1α overexpression. VEGF has been studied in many human neoplasms, such as lung, breast, gastrointestinal tract, renal, and ovarian carcinomas [24–28]. A humanized anti-VEGF monoclonal antibody (bevacizumab; Avastin) has been approved as a first-line treatment for metastatic colorectal cancer in humans [29]. In our case, VEGF was positive in 77.8% (56/72) of the FMGCs. As noted above, HIF-1α and VEGF overexpressions in FMGCs are not uncommon.

The positive signals of HIF-1α were diffusely distributed throughout the FMGC or restricted to perinecrotic neoplastic cells, consistent with the pattern of HIF-1α in human breast cancers [30]. In addition, our results of univariate analysis (Kaplan-Meier) demonstrated that HIF-1α overexpression was significantly correlated with a lower overall survival rate and considered a potential prognostic factor in FMGCs. This finding is consistent with previous studies of human breast cancers [31–35]. In this study, we used a threshold (> 0% positive nuclei/5 representative high power fields) for HIF-1α that was different from the thresholds (> 1%, or > 10% positive nuclei/5 representative high power fields) used in previous studies conducted in humans [30, 31, 36–39]. Furthermore, there is no significant association between overall survival rate and HIF-1α overexpression in FMGCs if the thresholds for HIF-1α (> 1%, or > 10% positive nuclei/5 representative high power fields) are used. According to this finding, > 0% is considered an optimal prognostic threshold for FMGCs. VEGF overexpression was not found to be significantly correlated with overall survival rate in this study, which is consistent with some of the previous studies of FMGCs [16–19]. A previous study has demonstrated that VEGF overexpression is significantly correlated with clinical outcome [16]. However; the prognostic threshold for VEGF is 72.1%, which is not a conventional method to evaluate the result of VEGF expression [16]. Therefore, a more conventional scoring principle to evaluate VEGF expression, one used in recent studies in FMGCs and human breast cancers, was chosen in this study [19, 40].

The correlation between HIF-1α and VEGF overexpressions was not significant in the present study. Previous studies have suggested that HIF-1α overexpression can occur in an early stage of breast cancer (before the presence of angiogenesis or invasion) [8, 36]. Therefore, it is speculated that the regulation of HIF-1α and VEGF expressions in FMGCs may be more complicated than or different from that in humans. Although the expression of VEGF is up-regulated during hypoxia by HIF-1α, the significant correlation between their IHC stainings is still controversial in previous human studies of gastric cancer, non-small cell lung cancer, and esophageal carcinoma [41–43]. They have pointed out that factors other than HIF-1α can regulate VEGF expression [41–43]. Many factors such as hormones, cytokines and cellular stress can regulate VEGF transcription as well [44].

The advantages of using TMAs include cost-effectiveness and rapid simultaneous molecular profiling of tissue samples from large cohorts. However, few studies have used TMAs in feline tissue [45, 46]. The weakness of TMAs is the relatively small size of samples, and thus the representativeness may vary among different samples. TMAs can be a useful method to detect VEGF expression in FMGCs because it tends to be large-scale. However, the use of TMAs for evaluating the HIF-1α expression in FMGCs may be limited due to the low expression level and the restrictive tissue distribution (such as perinecrotic tissue) of HIF-1α. Most importantly, TMAs are usually constructed by taking the most representative areas without marked necrosis or inflammation to reserve more neoplastic cells, and it may conflict with the expression pattern of HIF-1α, which is usually perinecrotic distribution. Therefore, the whole tissue sections of 38 cats with 1-year follow-up were used for investigating the correlation between HIF-1α overexpression and overall survival rate.

Several prognostic indexes for FMGCs have been advanced in previous studies, including clinical stage, tumor grading, and proliferation markers [5, 47]. In general, veterinary pathologists follow the World Health Organization morphological classification of feline mammary tumor to categorize the histologic types of FMGCs, but no significant correlation between histologic type and prognosis has been indicated, except the invasive micropapillary carcinoma [48, 49]. A previous study has demonstrated that invasive micropapillary carcinomas have a distinct histologic pattern with more aggressive biological behavior and poor clinical outcome [49]. Although this statement cannot be supported in this study due to the small sample size (*n* < 5), the cats with invasive micropapillary carcinomas were all dead within 1 year, and thus the 1-year overall survival rate was 0%.

The EE grading system is a gold standard for the histologic grading of human breast cancers, but it may be of limited utility in FMGCs [50]. The Revised EE (REE) and the novel grading system for FMGC have been advanced for their prognostic value of histologic grading [50]. Although the REE grading system is prognostic for FMGCs, no significant correlation between the novel grading system and overall survival rate was indicated in our study. Therefore, it is presumed that the correlation between pathological factors and prognosis may be different in different countries and feline breeds. Therefore, to determine or predict the prognosis of cats with FMGCs, all clinical/pathological parameters should be of concern.

In conclusion, this study suggests that the overexpression of HIF-1α indicates poor prognosis in cats with FMGCs. HIF-1α might therefore be a useful biomarker for feline cancer therapy and prediction of the clinical outcome, and thus developing compounds that inhibit HIF-1α may be a potential approach to FMGCs treatment. In addition, there are some limitations in this study. Two major concerns are 1) the sample size in this study is relatively small. 2) the nature of this study is retrospective and our results are still preliminary. Thus, further investigations on the mRNA expression levels of HIF-1α and VEGF by RNAscope® technology and other molecules involved in their upstream and downstream of the signaling pathway based on an bigger sample size are warranted to determine the potential molecular pathogenesis of FMGCs.

## Conclusions

This is the first study that investigates the expression and the prognostic significance of HIF-1α in feline mammary carcinomas. The results showed that overexpression of HIF-1α was significantly correlated with the overall survival rate, whereas there was no significant correlation between VEGF overexpression and overall survival rate. Given the major role of HIF-1α in compensating for oxygen deficiency by increasing availability of oxygen or providing metabolic adaptation of tumor cells, the inhibition of this activity might provide a potential approach to FMGC treatments.

## Methods

The FMGCs used in this study were obtained from the archives of the Graduate Institute of Molecular and Comparative Pathobiology, National Taiwan University. The samples were surgically obtained by mastectomy or block dissection from cats of different breeds (*n* = 72). Formalin-fixed and paraffin wax-embedded tissue blocks were prepared and available as hematoxylin and eosin (HE)-stained sections. Clinical data including age, reproductive status, and clinical stage were obtained [51]. All of the FMGC cases included in the study were followed up for at least 1 year after surgery by the referring veterinary surgeons, and the post-operative courses of FMGCs were evaluated. FMGC cases with follow-up were then grouped into 2 distinct outcomes: (1) alive, and (2) tumor-related death/euthanasia. Other conditions, such as lost to follow-up, non-tumor-related death/euthanasia or death/euthanasia of undetermined causes, were excluded in this study.

### Histological examination

Histologic slides were reviewed independently by an assessor, with direct assistance from an experienced veterinary pathologist (Chian-Ren Jeng). FMGCs were classified by different histological patterns, including tubulopapillary, solid, cribriform and micropapillary invasive [49]. In the FMGCs presenting more than one histologic pattern, the most predominant pattern in the tissue sections was chosen.

The histologic grading of FMGCs was determined by using the EE, REE, and novel grading systems [50, 52]. Other information, such as central necrosis, squamous differentiation, stromal response, lymphovascular invasion and the presence of lymph node metastasis, were also documented. Briefly, necrosis and squamous differentiation were classified as 2 separate categories. Necrotic areas of the neoplasm with more than 25% of necrotic areas were classified as “extensive”, while other conditions were regarded as “no to mild”. Stromal response was classified subjectively as follows: (1) none to mild, (2) peritumoral, and (3) intratumoral. Changes consistent with squamous differentiation included at least 2 of the following: decreased N:C ratio, increased cytoplasmic eosinophilia, increased cell size, and increased angularity of cell margin [50]. Squamous differentiation was classified according to proportion of metaplastic cells in all neoplastic cells and divided in 2 subgroups as (1) ≤ 5%, (2) > 5%. A tumor was positive for lymphovascular invasion if neoplastic emboli were clearly seen within an endothelium-lined lymphatic and/or vascular lumens.

### Tissue microarray (TMA) construction

TMAs were prepared by Array Biotechnology Co., Ltd. Cylindrical cores of 1.5 mm in diameter were taken from tumor areas of paraffin blocks and inserted into the TMA block. Three tissue arrays were constructed at the same time and composed of the most representative areas without marked necrosis and/or inflammation [45]. Tumor emboli and metastatic tumors in lymph nodes were also included. The TMA blocks were sectioned at 4 μm for subsequent IHC study.

### IHC stainings of HIF-1α and VEGF

IHC analysis was performed by using anti-HIF-1α (Clone: H1alpha67; diluted 1:200; Abcam, Cambridge, UK) and VEGF antibody (Clone: A-20; diluted 1:100; Santa Cruz Biotechnology, Inc., CA), which had good cross-reactivity with feline tissues [3, 40]. Tissue sections were deparaffinized in xylene, rehydrated in a graded series of ethanol solutions (100, 100, 95, and 80%), and then microwaved at 95 °C for 45 min for HIF-1α and 30 min for VEGF in commercial trilogy (Cell Marque, California, USA) for antigen retrieval, and then allowed to cool for 20 min. Slides were incubated in 10% nonimmune goat serum (Dako Ltd., Ely, UK) diluted by 1X PBS at room temperature for 30 min. The tissue sections were incubated with anti-HIF-1α and anti-VEGF antibodies respectively for 1.5 h and 1 h at room temperature in a moist chamber. Endogenous peroxidase activity was blocked with a solution of 3% H_2_O_2_ in methyl alcohol for 10 min. The slides were incubated with secondary antibody REAL Envision Detection System Peroxidase/DAB+, mouse/rabbit (Dako), for 60 min according to the manufacturer’s instructions. The slides were then stained with 3–3′-diaminobenzide tetrahydrochloride for 3 min. Sections were counterstained with Mayer’s hematoxylin solution. 1 X TBST was used as washing buffer in the procedure of IHC. Negative control included incubation with nonimmune goat serum instead of primary antibody. Feline tissue known to react with HIF-1α and human invasive breast carcinoma known to express VEGF were used as positive controls. IHC stainings of HIF-1α and VEGF were conducted in TMAs of 72 FMGC cases and whole sections of 38 FMGC cases with 1-year follow-up.

### Scoring criteria

The positive signals of IHC stainings for both HIF-1α and VEGF were scored by using the previous criteria [19, 40]. HIF-1α was defined as overexpressed when there were any neoplastic cells with nuclear positivity. The presence of cytoplasmic positivity of HIF-1α in neoplastic cells was not indicative. The positive signals of VEGF were mainly observed in the cytoplasm of neoplastic cells, and the score was ascertained by consideration of both staining density and intensity as follows: 0, a complete lack of staining; 1, < 50% positive neoplastic cells with weak staining; 2, weak positive staining in ≥50% of neoplastic cells or strong staining in < 50% of cells; 3, strong positive staining in ≥50% of neoplastic cells. VEGF overexpression is defined as a score of 2 and 3, while VEGF non-overexpression is defined as a score of 0 or 1.

### Statistical analyses

Statistical analyses were all performed in Prism (GraphPad Sofware, CA, USA) and IBM SPSS statistics version 22.0 (IBM Corp., Armond, NY). The correlation between HIF-1α/VEGF overexpressions and clinical/pathological factors were performed using chi-square tests. Spearman correlation from ranks was used to analyze the correlation between VEGF and HIF-1α. Curves for 1-year overall survival rate were estimated with Kaplan-Meier analysis, and differences between 2 groups were evaluated using the log-rank test for univariate survival analysis. A *p* value < 0.05 was considered statistically significant.

## Data Availability

The datasets generated and/or analyzed during the current study are available from the corresponding author on reasonable request. For more information, please contact the corresponding authors.

## Abbreviations

FMGC: Feline mammary gland carcinoma
HIF-1α: Hypoxia-inducible factor 1 alph
VEGF: Vascular endothelial growth factor
TMA: Tissue Microarray
IHC: Immunohistochemical
WHO: World Health Organization.
